# Prostaglandins differentially modulate mucosal‐associated invariant T‐cell activation and function according to stimulus

**DOI:** 10.1111/imcb.12617

**Published:** 2023-02-18

**Authors:** Hema Mehta, Irene Tasin, Carl Philipp Hackstein, Christian Willberg, Paul Klenerman

**Affiliations:** ^1^ The Peter Medawar Building for Pathogen Research University of Oxford Oxford UK; ^2^ NIHR Biomedical Research Centre University of Oxford Oxford UK

**Keywords:** innate‐like T‐cell, mucosal‐associated invariant, prostaglandins, T‐cells

## Abstract

Mucosal‐associated invariant T (MAIT) cells are an *innate*‐like T‐cell type conserved in many mammals and especially abundant in humans. Their semi‐invariant T‐cell receptor (TCR) recognizes the major histocompatibility complex–like molecule MR1 presenting riboflavin intermediates associated with microbial metabolism. Full MAIT cell triggering requires costimulation *via* cytokines, and the cells can also be effectively triggered in a TCR‐independent manner by cytokines [e.g. interleukin (IL)‐12 and IL‐18 in combination]. Thus, triggering of MAIT cells is highly sensitive to local soluble mediators. Suppression of MAIT cell activation has not been well explored and could be very relevant to their roles in infection, inflammation and cancer. Prostaglandins (PG) are major local mediators of these microenvironments which can have regulatory roles for T cells. Here, we explored whether prostaglandins suppressed MAIT cell activation in response to TCR‐dependent and TCR‐independent signals. We found that protaglandin E_2_ (PGE_2_) and to a lesser extent protaglandin D_2_ (PGD_2_), but not leukotrienes, suppressed MAIT cell responses to *Escherichia coli* or TCR triggers. However, there was no impact on cytokine‐induced triggering. The inhibition was blocked by targeting the signaling mediated *via* PG receptor 2 (PTGER2) and 4 (PTGER4) receptors in combination. These data indicate that prostaglandins can potentially modulate local MAIT cell functions *in vivo* and indicate distinct regulation of the TCR‐dependent and TCR‐independent pathways of MAIT cell activation.

## Graphical abstract

Mucosal‐associated invariant T (MAIT) cell triggering requires costimulation *via* cytokines, and the cells can also be effectively triggered in a T‐cell receptor–independent manner by cytokines [e.g. interleukin (IL)‐12 and IL‐18 in combination]. Thus, triggering of MAIT cells is highly sensitive to local soluble mediators. Suppression of MAIT cell activation has not been well explored and could be very relevant to their roles in infection, inflammation and cancer. Prostaglandins are major local mediators of these microenvironments; here we show they can differentially modulate MAITs dependent on the stimulus.

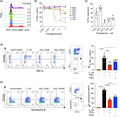

## INTRODUCTION

Mucosal‐associated invariant T (MAIT) cells are a subset of T cells with innate and effector‐like phenotype. Unlike conventional T cells, which are restricted by highly polymorphic major histocompatibility complex molecules, MAIT cells recognize riboflavin‐derivative antigens presented by the evolutionary conserved MR1 molecule.^1^ MAIT cells make up a significant proportion of the T cells in the blood in humans and are also enriched in several peripheral organs.^2^ MAIT cells respond to stimulation by MR1‐presented ligands *via* their T‐cell receptor (TCR) or by cytokines alone.^3^, ^4^ The sensitivity of MAIT cells to their local environment allows them to exhibit distinct functional responses depending on the presence of soluble mediators.^5^, ^6^

Eicosanoids are produced during inflammation and are involved in the immune response, eliciting broad effects. Of the main eicosanoids, prostaglandins (PGs) are key players differentially regulating immune responses and are a group of commonly reported metabolites that are significantly increased in cancers.^7^

PGs are bioactive lipids that are readily synthesized by the cyclooxygenase‐1– or cyclooxygenase‐2–inducible enzymes from arachidonic acid in response to numerous triggers that include proinflammatory and mitogenic stimuli.^8^ The constitutive cyclooxygenase‐1 expression allows prostagladin E2 (PGE_2_) to exert its homeostatic and immune surveillance functions while the cyclooxygenase‐2 isoform is rapidly induced in response to injury or infection. PGE_2_ is the most abundant PG but like all PGs has a short half‐life and its metabolic turnover is regulated by 15‐hydroxyprostaglandin dehydrogenase, an enzyme that can breakdown PGE_2_.^9^ PGE_2_ is largely known for its tumor‐promoting role whereby it suppresses immune cells, particularly cytotoxic T cells (cytotoxic T lymphocytes).^10^ In addition to the anti‐inflammatory roles of PGE_2_, such as suppression of interleukin (IL)‐2, IL‐12 and interferon‐gamma (IFN‐γ), PGs also play an important role during the acute phase of inflammation where it synergizes with proinflammatory mediators such as tumor necrosis factor‐α (TNF‐α) and transiently promotes dendritic cell maturation by upregulating CCR7 (C–C chemokine receptor type 7).^11^ Some other functions of PGE_2_ are the induction of type‐2 and type‐17 immune cells, inducing IL‐10^+^ CD4^+^ T regulatory cells and indirectly regulating wound healing by promoting differentiation of M2 macrophages to accelerate tissue repair.^12^, ^13^, ^14^, ^15^

The role of prostaglandins, in particular PGE_2_, has been reviewed in various pathological settings^16^, ^17^ and because MAIT cells have been studied in the same settings, we speculated that the increased prostaglandin levels could impact MAIT cell function. Our study aimed to investigate the impact of inflammatory mediators (prostaglandins and leukotrienes) on MAIT cell activation and function *in vitro*. We hypothesized that prostaglandins would have an inhibitory effect on MAIT cell activation and function. As MAIT cells can be activated independently of their TCR, a second hypothesis was that PGE_2_ would impact only the TCR‐dependent triggering, not the TCR‐independent triggering.

## RESULTS

### Prostaglandins inhibit the proinflammatory capacity of antigen‐stimulated MAIT cells in a concentration‐dependent manner

**Figure 1:**
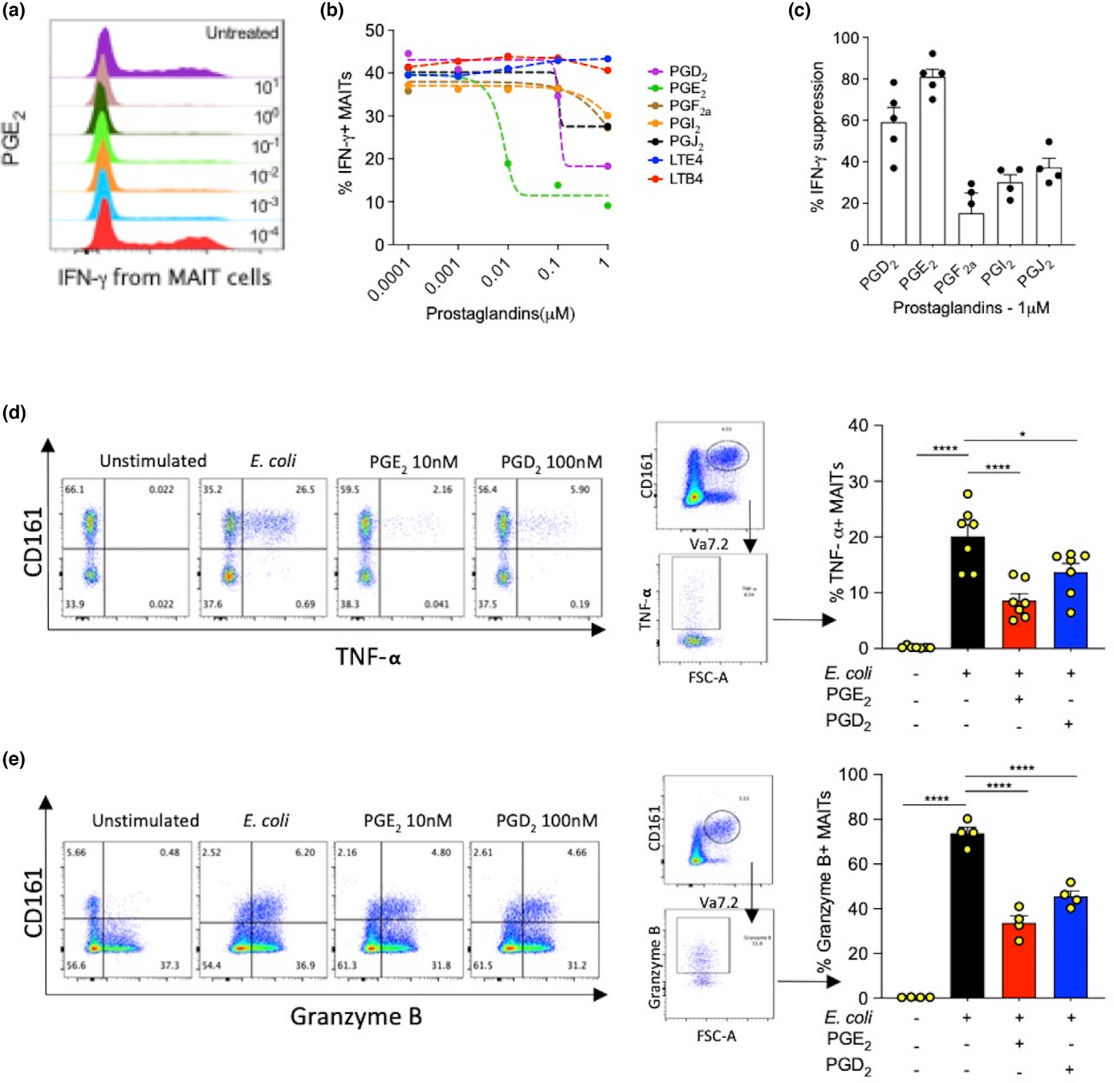
Prostaglandin potency and modulation of mucosal‐associated invariant T (MAIT) cell proinflammatory and cytolytic functions. CD8^+^ T cells were isolated from healthy donor peripheral blood mononuclear cells (PBMCs), stimulated with *Escherichia coli*–activated THP‐1 cells ± PGs/LTs for 16 h followed by analysis of CD8^+^CD161^++^Vα7.2^+^ MAIT cells by flow cytometry. **(a)** Representative histogram plot showing the dose‐dependent suppression of interferon‐gamma (IFN‐γ) from antigen‐stimulated CD8^+^ MAIT cells. **(b)** Prostaglandins and leukotrienes titrated onto antigen‐stimulated MAIT cells. IFN‐γ expression from MAIT cells treated with prostaglandins or leukotrienes, 100 pM to 1 μM (*n* = 5). **(c)** Potency of prostaglandins expressed as percentage IFN‐γ suppression from THP‐1+ *E. coli*–stimulated CD8^+^ MAIT cells ± PGs. Percentage IFN‐γ suppression calculated as 100−(treated/untreated × 100) (*n* = 5). **(d)** Illustrative fluorescence‐activated cell sorting (FACS) plots of CD8^+^CD161^++^ T cells, largely MAIT cells (left); representative flow plots used for quantification of tumor necrosis factor‐α (TNF‐α) expression from MAIT cells (CD8^+^CD161^++^Vα7.2^+^; middle) and CD8^+^‐MAIT cell TNF‐α expression quantification bar chart (right; *n* = 7). **(e)** Illustrative FACS plots of CD8^+^CD161^++^ T cells, largely MAIT cells (left); representative flow plots used for quantification of granzyme B expression from MAIT cells (CD8^+^CD161^++^Vα7.2^+^) (middle) and CD8^+^‐MAIT cell granzyme B expression quantification bar chart (right; *n* = 4). Statistical significance: one‐way ANOVA, Šidák's multiple comparisons test, comparison between stimulated (untreated) and prostaglandin‐treated MAIT cells, *P < 0.05, ****P < 0.0001. FSC‐A, forward scatter–area; LT, leukotriene; LTB_4_, leukotriene E_4_; LTE_4_, leukotriene E_4_; PG, prostaglandin.

To investigate the effect of prostaglandins on MAIT cell activation and function, we titrated five different prostaglandins into our antigen stimulation model, comprising *Escherichia coli*–stimulated THP‐1 cell activation of purified CD8^+^ T cells. In addition to testing prostaglandins, we tested the effects of two major leukotrienes on MAIT cell activation and function as they are also found at sites of MAIT cell enrichment such as the lung and colon.^18^, ^19^ Synthetic prostaglandins were added at the beginning along with *E. coli* and cells were cocultured for 16 h prior to analysis by flow cytometry; details of flow cytometry gating of MAIT cells are available in Supplementary figure 1. All five prostaglandins showed some degree of suppression on CD8^+^ MAIT cell IFN‐γ expression at high concentrations, but not at concentrations lower than 10 nM (Figure 1b). Of the five prostaglandins tested, PGE_2_ was the most potent followed by prostaglandin D_2_ (PGD_2_) and the remaining prostaglandins were mildly suppressive at concentrations above 100 nM (Figure 1b, c). In contrast to prostaglandins, the related leukotrienes did not have a suppressive effect on MAIT cell–IFN‐γ expression, even at high concentrations (Figure 1b). To compare the relative suppressive capacity of prostaglandins to each other we measured the suppressive potential of the prostaglandins at 1 μM concentration, which always produced a suppressive effect for all five prostaglandins (Figure 1c). Again, PGE_2_ was most potent, showing on average 90% suppression. Prostaglandin F_2a_ (PGF_2a_) was the least potent prostaglandin, but still suppressed approximately 30% of the IFN‐γ expression from antigen‐stimulated CD8^+^ MAIT cells (Figure 1c). The suppression of effector molecule expression from MAIT cells was not restricted to IFN‐γ. PGD_2_ and PGE_2_ could also significantly suppress the expression of TNF‐α and granzyme B expression from MAIT cells (Figure 1d, e).

### PGE_2_ inhibits IFN‐γ production by antigen‐stimulated MAIT cells but not in cytokine‐stimulated MAIT cells

**Figure 2:**
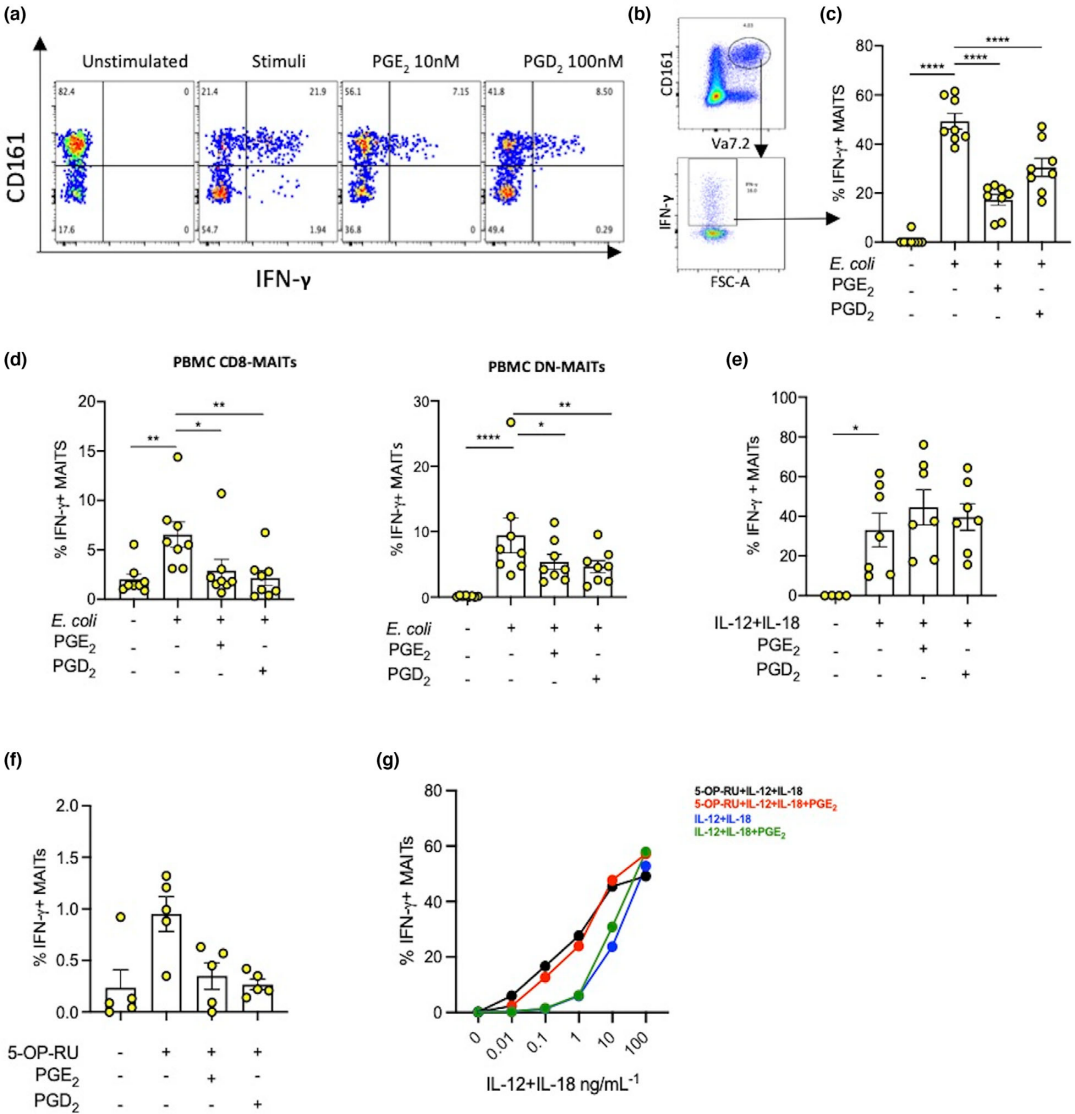
Impact of PGD_2_ and PGE_2_ on different mucosal‐associated invariant T (MAIT) cell stimulation pathways. Interferon gamma (IFN‐γ) expression was measured by flow cytometry from MAIT cells stimulated using antigen or cytokines and treated with 100 nM PGD_2_ and 10 nM PGE_2_ for 16 h. **(a)** Illustrative fluorescence‐activated cell sorting plot of IFN‐γ expression from CD8^+^CD161^++^ T cells, largely MAIT cells. **(b)** Representative flow plots used for quantification of IFN‐γ expression from CD8^+^CD161^++^Vα7.2^+^ MAIT cells. **(c)** CD8‐MAIT cell IFN‐γ expression quantification bar chart (*n* = 8). **(d)** IFN‐γ expression from CD8‐MAIT and double‐negative (DN)‐MAIT populations from healthy donor peripheral blood mononuclear cells (PBMCs) stimulated with *Escherichia coli* and treated ± PGD_2_/PGE_2_ (*n* = 8). **(e)** IFN‐γ expression from cytokine‐stimulated healthy donor purified CD8‐MAIT cells (*n* = 7). **(f)** IFN‐γ expression from ligand‐stimulated CD8‐MAIT cells (*n* = 5). **(g)** % IFN‐γ expression from PBMC–MAIT cells stimulated with ligand alone or ligand and cytokines ± PGE_2_ (*n* = 8). Statistical significance: one‐way ANOVA, Šidák's multiple comparisons *t*‐test, comparison between stimulated (untreated) and prostaglandin‐treated MAIT cells, *P < 0.05, **P < 0.01, ****P < 0.0001. 5‐OP‐RU, 5‐(2‐oxopropylideneamino)‐6‐d‐ribitylaminouracil; FSC‐A, forward scatter–area; IL, interleukin. PG, prostaglandin.

As MAIT cells can be activated by multiple pathways,^4^, ^20^ we tested the effect of prostaglandins on MAIT cells activated by antigen, cytokines or with the help of other leukocytes. Stimulation of MAIT cells within the peripheral blood mononuclear cell (PBMC) pool [i.e. activation *via* primary blood antigen‐presenting cells (APCs)] produced less IFN‐γ as compared with purified CD8^+^ MAIT cells stimulated using *E. coli* and THP‐1 cells (Figure 2c, d). Double‐negative PBMC–MAITs cells also responded similarly to PBMC–CD8–MAITs (Figure 2d). Notwithstanding the difference in stimulation potential, both PGD_2_ and PGE_2_ suppressed MAIT cell IFN‐γ significantly, where they reduced IFN‐γ expression by 50% or more (Figures 1c and 2c, d). In contrast to the suppressive effect of PGs seen for both purified (Figure 2c) and unpurified (Figure 2d) antigen‐stimulated MAIT cells, PGs did not suppress IFN‐γ production in cytokine‐stimulated (Figure 2e) CD8^+^ MAIT cells. Similar effects to the *E. coli* antigen stimulation model were observed for the pure ligand stimulation of both purified CD8 T‐cell MAITs (Figure 2f), whereas if the MAIT‐activating cytokines IL‐12 and IL‐18 are present in combination with the pure ligand, then PGE_2_ does not suppress MAIT cell IFN‐γ (Figure 2g). MAIT IFN‐γ expression was also not suppressed by prostaglandin following stimulation by ligand and cytokines over a longer time course of 72 h (Supplementary figure 2).

### PGE_2_ signals to MAIT cells through EP2/EP4

**Figure 3:**
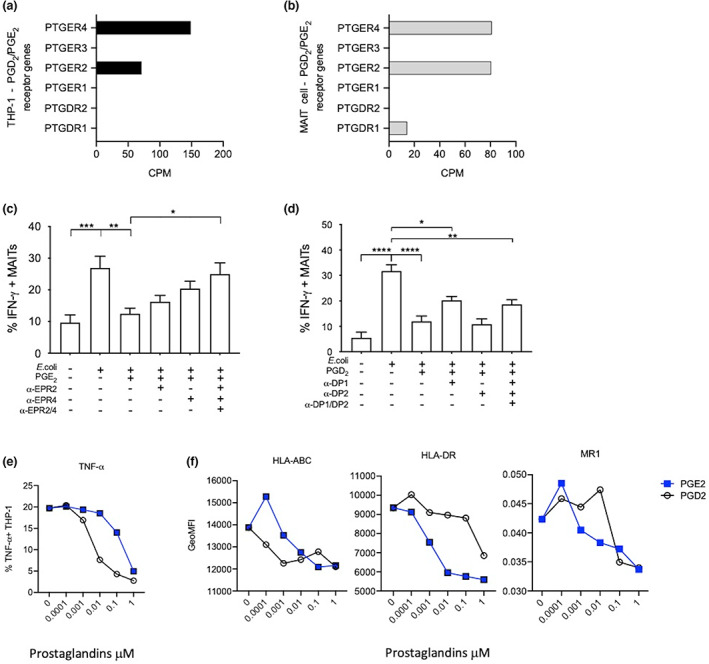
Prostaglandin receptor expression profile and receptor signaling blockade in THP‐1 and mucosal‐associated invariant T (MAIT) cells. Receptor gene expression on THP‐1 cell lines and MAIT cells analyzed using bulk RNA‐seq expression data sets. Raw gene expression counts for PGD_2_ receptors PTGDR1 and PTGDR2, and PGE_2_ receptors PTGER1–4 expressed as counts per million (CPM) in **(a)** the THP‐1 cell line and **(b)** CD3^+^CD161^++^Vα7.2^+^–sorted MAIT cells derived from healthy donor blood. CD8 T cells isolated from healthy donor peripheral blood mononuclear cells (PBMCs) were stimulated with *Escherichia coli*–activated THP‐1 cells ± receptor antagonist for 16 h followed by analysis of CD8^+^CD161^++^Vα7.2^+^ MAIT cells by flow cytometry. **(c)** Interferon gamma (IFN‐γ) expression from CD8 MAIT cells treated with 10 nM PGE_2_ and 1 μM EP2 and EP4 receptor antagonist (*n* = 8). **(d)** IFN‐γ expression from CD8 MAIT cells treated with 100 nM PGD_2_ and 1 μM DP1 and DP2 receptor antagonist (*n* = 11). THP‐1 cells stimulated with *E. coli* with or without ± PGE_2_ and PGD_2_. **(e)** % Tumor necrosis factor‐α (TNF‐α). **(f)** Geometric mean fluorescent intensity of HLA‐ABC, HLA‐DR and MR1 on THP‐1 cells measured by flow cytometry (*n* = 3). Statistical significance: one‐way ANOVA, Šidák's multiple comparisons *t*‐test, comparison between stimulated (untreated) and PG‐treated MAIT cells, PG‐treated and receptor antagonist or untreated and receptor antagonist, *P < 0.05, **P < 0.01, ***P < 0.001, ****P < 0.0001, ns, nonsignificant. HLA, human leucocyte antigen; PG, prostaglandin; EP, prostaglandin E receptor subtype; EPR, prostaglandin subtype E receptor; DP, prostaglandin D receptor subtype.

To determine the receptors used by prostaglandins to signal to MAIT cells, we looked at the gene expression in the THP‐1 cell line and MAIT cells from existing RNA‐seq data.^21^ We found that, of the four PGE_2_ receptors (PTGER1‐4), PTGER2 and PTGER4 were expressed at the messenger RNA level in both cell types. THP‐1 cells had greater PTGER4 receptor gene expression compared with PTGER2, whereas it was equal in MAIT cells (Figure 3a). Neither PTGER1 nor PTGER3 were detected on THP‐1 or MAIT cells (Figure 3a). PGD_2_ has two known receptors, prostagladin D receptor 1(PTGDR1) and 2 (PTGDR2); of these two receptors PTGDR1 was expressed only on MAIT cells (Figure 3b).

To further understand the signaling mechanism of prostaglandins, we used PGE_2_ and PGD_2_ receptor antagonists. Using the PTGER2 and PTGER4 receptor antagonists individually resulted in limited reversal of PGE_2_‐mediated suppression of IFN‐γ in MAIT cells. Whereas, in combination, the receptor antagonists were able to completely prevent PGE_2_‐mediated suppression (Figure 3c). By contrast, blockade of the PTGDR1 alone or in combination with PTGDR2 resulted in limited blockade of PGD_2_‐mediated suppression of IFN‐γ by MAIT cells (Figure 3d). The latter confirms the lack of PTGDR2 expression on MAIT and THP‐1 cells and possible expression of other PGD_2_ receptors. The limited effect of receptor DP1 and DP2 receptor antagonism may be attributed to secondary metabolite/receptor signaling to MAIT cells by PGD_2_ degradation metabolites, for example, delta‐PGD_2_ and prostagladin J2 (PGJ_2_).^22^

### Indirect impact of PGE_2_ mediated through the APCs

While the aforementioned experiments indicate that PGE_2_ and PGD_2_ can act directly on T cells in the absence of an APC, we also addressed whether there could be an additional impact *via* the suppression of MR1 or inhibition of MAIT costimulatory cytokines such as IL‐12, IL‐18 and TNF‐α. We measured the expression of antigen‐presenting molecules on the THP‐1 cell line treated with PGD_2_ and PGE_2_ by flow cytometry. Our data show a dose‐dependent decrease in the expression of several antigen‐presenting molecules, MR1 as well as major histocompatibility complex class I and major histocompatibility complex class II, while the proinflammatory cytokine TNF‐α was also suppressed by both PGE_2_ and PGD_2_ (Figure 3e, f). IL‐12 and IL‐18 expression was difficult to measure by flow cytometry or ELISA, so we used qPCR to determine PG effects on their gene expression. The results did not indicate any clear impact associated with dose titration of PGs on these cytokines (Supplementary figure 3).

### Prostaglandins can act directly on MAIT cells suppressing their TCR‐mediated activation

**Figure 4:**
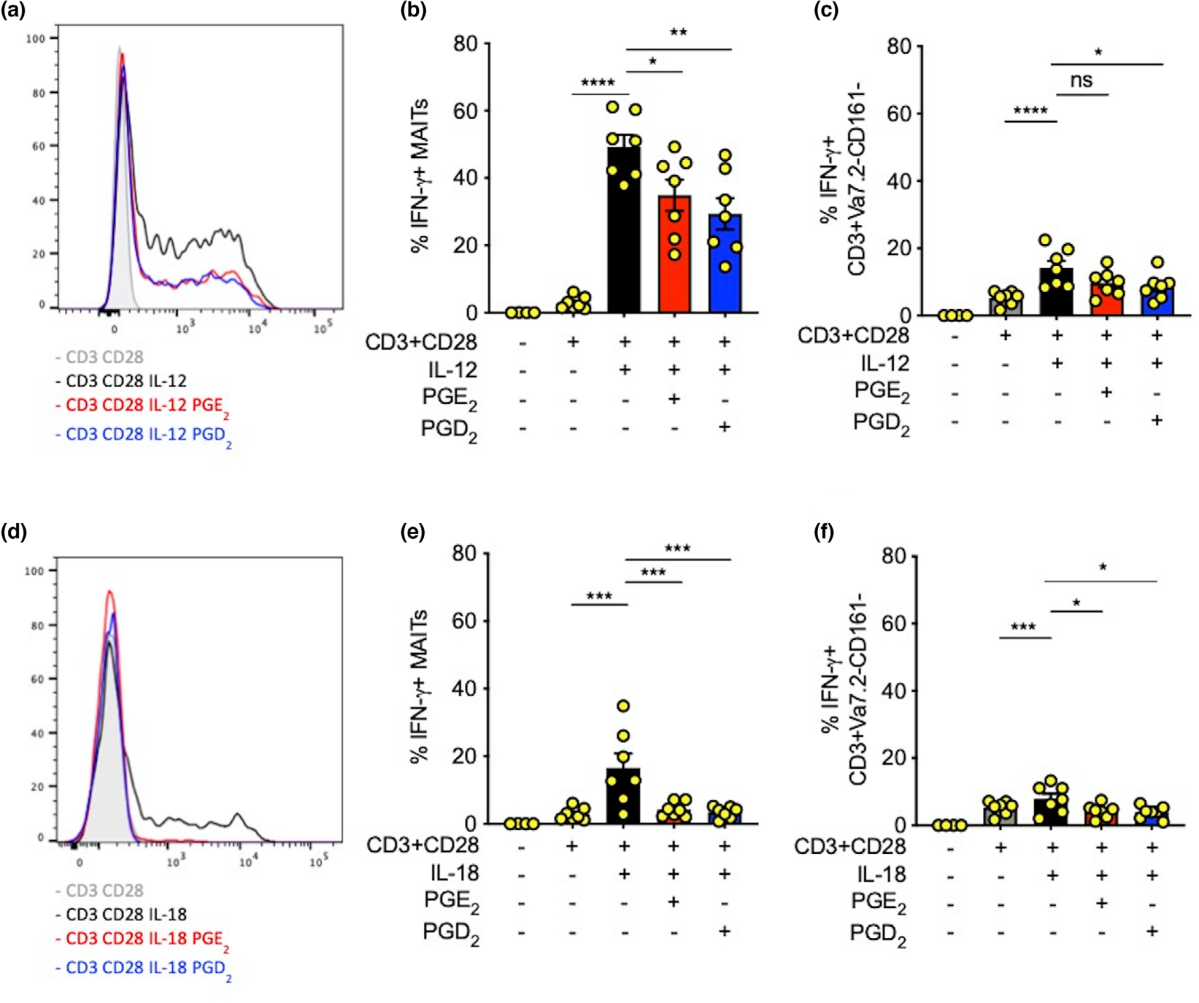
Impact of prostaglandins on T‐cell receptor (TCR) + cytokine–stimulated mucosal‐associated invariant T (MAIT) cells *versus* non‐MAIT cell CD161‐negative populations. CD8 T cells isolated from healthy donor peripheral blood mononuclear cells were stimulated with plate‐bound CD3, soluble CD28 (TCR) and interleukin (IL)‐12 ± PGs for 24 h followed by flow cytometry analysis. **(a)** Representative overlay histogram showing interferon‐gamma (IFN‐γ) expression in TCR + interleukin (IL)‐12–stimulated MAIT cells treated with prostaglandin D2/E2 (PGD_2_/PGE_2_). **(b)** Quantification of IFN‐γ expression from CD8^+^ MAIT cells (*n* = 7) and **(c)** CD161‐negative T‐cell compartment stimulated with TCR + IL‐12 and treated with PGD_2_/PGE_2_ (*n* = 7). **(d)** Representative overlay histogram showing IFN‐γ expression in TCR + IL‐18–stimulated MAIT cells treated with PGD_2_/PGE_2_. **(e)** Quantification of IFN‐γ expression from TCR + IL‐18–stimulated CD8^+^ MAIT cells (*n* = 7). **(f)** CD161‐negative T‐cell compartment stimulated with TCR + IL‐18 and treated with PGD_2_/PGE_2_ (*n* = 7). Statistical significance: one‐way ANOVA, Šidák's multiple comparisons *t*‐test, comparison between stimulated (untreated) and PG‐treated MAIT cells, *P < 0.05, **P < 0.01, ***P < 0.001, ****P < 0.0001, ns, nonsignificant; PG, prostaglandin.

As MAIT cells express PGD_2_ and PGE_2_ receptor genes (Figure 3b), we decided to investigate the direct effect of PGs on MAIT cells using APC‐free models (i.e. to exclude any effects of the PGs on APC function). In the TCR‐independent cytokine stimulation model the PGs did not suppress the stimulation (Figure 2e). In the TCR‐dependent stimulation model using plate‐bound CD3 and a soluble CD28^+^ cytokine, either IL‐12 or IL‐18 (alone, but not in combination), we saw substantial suppression of MAIT–IFN‐γ production in the presence of PGE_2_ or PGD_2_ (Figure 4a, b, d, e). The effects of PG were similar for both IL‐12 and IL‐18 costimulation of the TCR pathway, but the IL‐18 costimulation induced IFN‐γ production from MAIT cells to a lower extent than IL‐12 (Figure 4b, d). These data indicate that the PG effect can be mediated directly on MAIT cells, even if APCs are also potentially impacted. Even though a lower proportion of Vα7.2^−^CD161^−^ were induced to express IFN‐γ in response to TCR + IL‐12 or TCR + IL‐18, PGs still reduced the IFN‐γ response by this population to a significant extent (Figure 4c, f).

### Analysis of tissue repair factors *in vitro*

Of note, even though PGs are implicated in tumor progression^17^, ^23^ with a strong suppressive capacity, in the steady state PGE_2_ is involved in homeostasis and exhibits wound healing properties.^13^, ^24^ Likewise, MAITs have recently been shown to possess tissue repair function.^21^, ^25^, ^26^ We attempted to explore the effect of PGE_2_ on MAIT wound healing functions using an extended coculture model where MAIT cells were stimulated with *E. coli* or 5‐OP‐RU [5‐(2‐oxopropylideneamino)‐6‐d‐ribitylaminouracil] and IL‐12 + IL‐18 ± exposure to PGE_2_. This model has been used previously to study the effects on tissue‐resident/mucosal MAIT cells.^21^ The tissue repair factors we measured by fluorescence‐activated cell sorting (CCL3, TNF‐α and furin) were not significantly enhanced nor suppressed. Only IL‐17F expression was modestly enhanced in the presence of PGE_2_, indicating a potentially favorable role for PGE_2_ modulation of MAIT cells for tissue repair (Supplementary figure 4). Data presented here and the role for PGE_2_ in MAIT‐dependent tissue repair overall require further study, ideally using tissue‐derived cells.

## DISCUSSION

MAIT cells are an innate‐like T‐cell population with potent and diverse functions. Studies of MAIT cells highlight their significance in different disease settings where they can play either protective or pathological roles. Our study demonstrates that MAIT cells can be modulated by PGs when activated *via* a TCR‐mediated signal. PGs negatively regulate MAIT cell proinflammatory and cytolytic activity which could be potentially important in tumor settings.

Prostaglandins and leukotrienes are produced by almost all cell types in the body; however, their effects are highly context dependent. As MAIT cells are dispersed throughout the body and because prostaglandins are present at sites of MAIT cell enrichment such as the liver and lung, we investigated the impact of PGs on MAIT cell activation and function. We saw a dose‐dependent suppression in MAIT cell activation by PGs but not by leukotrienes (Figure 1b). The difference in the modulatory capacity of some of the metabolites in these two groups of eicosanoids is likely to be dependent on their respective receptor expression on the APC and/or the MAIT cells. We identified PTGER2 and PTGER4 receptor gene expression in both MAIT cell and THP‐1 cells, whereas the PGD_2_ receptor PTGDR1 gene was only found in MAIT cells. As PTGDR1 is expressed only in MAIT cells and not in THP‐1 cells, this may support direct modulation of MAIT cells by PGD_2_, whereas PGE_2_ could indirectly modulate MAIT cells through APCs, or directly, as PGE_2_ receptor genes were expressed by both THP‐1 and MAIT cells. Further work using accurate antibody detection for these receptors (by fluorescence‐activated cell sorting or western blot) is required to definitively prove their expression on these cells. In addition to receptor signaling–mediated modulation,^27^ PGs have been shown to modulate several other stages of T‐cell activation,^28^ such as the phagocytosis of pathogens,^29^ and modulation of the APC cytokine expression profiles^30^, ^31^ leading to either inhibitory signaling or skewing^12^, ^23^ toward alternate phenotypes, particularly inducing regulatory T cells or T helper type 17, myeloid‐derived suppressor cells and M2 macrophages.^13^ Although we did not probe these aspects further in this study, our findings add to these in that we observe MAIT cells can be modulated directly, even in the absence of APCs.

Very high doses of prostaglandins were required to suppress MAIT cell proinflammatory cytokines and cytotoxic molecules in response to antigen stimulation (Figure 1). This effect, therefore, is likely to be seen in specific disease settings such as the tumor microenvironment where high levels of PGE_2_ are detected.^23^ As PGE_2_ can induce myeloid‐derived suppressor cells, it can be postulated that PGE_2_‐induced myeloid‐derived suppressor cells in the tumor microenvironment could also inhibit MAIT cell antitumor function. Other possible effects of PGE_2_ on MAIT cells could include the upregulation of checkpoint receptor PD‐1 (programmed cell death protein 1) in MAITs as shown for cytotoxic T lymphocytes,^32^ thereby further limiting their activation.

In contrast to the suppression of antigen‐stimulated MAIT cells, we observed sustained MAIT cell activation in the presence of PGE_2_ when MAIT cells were stimulated with the cytokines IL‐12 and IL‐18 in combination—the difference being most obvious using the APC‐free system. This highlights that these two activation pathways are not only functionally distinct but also differentially regulated. PGE_2_ may also prevent cytokine signaling to MAIT cells from the APCs by limiting their ability to make proinflammatory cytokines^33^ such as TNF‐α (Figure 3e). Compared with MAIT cells, PGE_2_ and PGD_2_ could also suppress the expression of IFN‐γ and TNF‐α in the Vα7.2^−^CD161^−^ T‐cell compartment, although the effect was less substantial, suggesting that MAIT cells are relatively more sensitive to PGs compared with conventional T cells.

Overall, MAIT cells are readily suppressed by prostaglandins (both directly and indirectly), but with differential impacts on TCR‐dependent and TCR‐independent activation. Limiting responses in a setting where the ligand is continuously present requires careful tuning—MAIT cells carefully adjust responses according to inflammatory cues. We can now incorporate prostaglandins into this list of cues, but the role of this in physiologic and pathologic conditions requires further study *in vivo*, including further analysis of a role in tissue homeostasis.

## METHODS

### Cells

Healthy donor blood leukocyte cones were obtained from NHS Blood and Transplant Service at the John Radcliffe Hospital, Oxford, under GI Biobank ethics 16/YH/0247. PBMCs were isolated from cones by density gradient centrifugation using Lymphoprep (STEMCELL Technologies, Cambridge, UK) following the manufacturer's protocol. THP‐1 cell lines were obtained from ECACC (UK) and maintained in RPMI‐1640 medium (Roswell Park Memorial Institute medium 1640) + 5% fetal bovine serum (Sigma Aldrich, Dorset, UK), 200 mM l‐glutamine (Gibco, Yorkshire, UK) and 100 μg mL^−1^ penicillin/streptomycin (Sigma Aldrich, Dorset, UK).

### *In vitro* assays

#### CD8 T‐cell isolation

CD8^+^ T cells were isolated from PBMCs using the CD8 purification kit (Miltenyi Biotec, Woking, UK) following the manufacturer's protocol.

#### Antigen stimulation of MAIT cells

THP‐1 cells (ECACC, Wiltshire, UK) were stimulated with paraformaldehyde‐fixed *E. coli* (DH5‐α, Invitrogen), 30 colony‐forming units per APC, and cocultured with isolated CD8^+^ T cells at a ratio of 1:2 APC to CD8^+^ T‐cell in a final volume of 200 μL per well. Stimulated MAIT cells were cultured ± prostaglandins PGD_2_ (Chem Cruz, Wembley, UK), PGE_2_ (Sigma Aldrich, Dorset, UK), PGF_2a_ (Cayman Chemicals, Michigan, USA), PGI_2_, PGJ_2_ (Cayman Chemicals, Michigan, USA), or leukotrienes LTB_4_ (leukotriene B_4_; Cayman Chemical, Michigan, USA), LTE_4_ (leukotriene E_4_; Enzo Life Science, Exeter, UK), or prostaglandin receptor antagonists SC51089‐EP1 antagonist (Tocris Bioscience, Bristol, UK), PF‐0441894 EP2 antagonist (Cayman Chemicals, Michigan, USA), L‐798106 EP3 antagonist (Cambridge Bioscience, Cambridge, UK), L161982 EP4 antagonist (Cayman Chemicals, Michigan, USA), BW868 DP1 antagonist and OC00459 DP2 antagonist (Cayman Chemicals, Michigan, USA) were added at 10 μM. The cells were cultured overnight at 37°C and 5% CO_2_ for 16 h. Brefeldin A (BFA; eBioscience, Cheshire, UK) and monensin (BioLegend, London, UK) were added for the final 4 h before staining cells.

#### Blocking assays

Receptor antagonists were added 30 min prior to the addition of prostaglandins to the antigen‐stimulation assay at a concentration of 1 μM.

#### PBMC stimulation

About 5 × 10^5^ PBMCs were stimulated with 25 colony‐forming units of paraformaldehyde‐fixed DH5‐α *E. coli* ± PGD_2_ (100 nM) or PGE_2_ (10 nM). PBMCs were cultured for 16 h at 37°C and 5% CO_2_. BFA/monensin was added for the final 4 h of the assay prior to staining.

#### Ligand stimulation

Purified CD8^+^ T cells were cultured with 5‐OP‐RU–stimulated THP‐1 cells ± 1 nM PGD_2_ or 10 nM PGE_2_ and cultured overnight. BFA and monensin were added for the final 4 h before staining cells.

PBMCs (5 × 10^5^) were stimulated ± 10 nM 5‐OP‐RU alone, or ± IL‐12 + IL‐18 cytokine titrations and treated ± 10 nM PGE_2_ for 72 h or overnight. BFA and monensin were added for the final 4 h before staining cells.

#### Cytokine stimulation of MAIT cells

Isolated CD8^+^ T cells were cultured ± 50 ng mL^−1^ IL‐12 or IL‐18 in a final volume of 200 μL ± 100 nM PGD_2_ or 10 nM PGE_2_ for 16 h. BFA and monensin were added for the final 4 h before staining cells.

#### TCR stimulation of MAIT cells

A 96‐well flat‐bottomed plate was coated with 50 μL of 2.5 μg mL^−1^ anti‐human‐CD3 antibody, clone HIT3 (BioLegend, London, UK) and incubated at 37°C for 2 h. The wells were washed two times with sterile phosphate‐buffered saline (PBS) and then replaced with 50 μL of culture medium containing 1 μg mL^−1^ CD28 antibody (BioLegend, London, UK). About 2 × 10^5^ healthy donor blood–purified CD8^+^ T cells were added to the wells ± IL‐12 or IL‐18 at 50 ng mL^−1^.

#### Extended coculture stimulation assay

About 1 × 10^6^ PBMCs were added to each well and stimulated with 30 colony‐forming units/cell *E. coli* or 5‐OP‐RU with 50 ng mL^−1^ IL‐12 + IL‐18 and incubated ± 10 nM PGE_2_ for 72 h. BFA and monensin were added for the final 4 h before staining cells.

#### Prostaglandin receptor gene counts

Gene counts were obtained from RNA‐seq data generated for THP‐1 cells (prepared in‐house) and sorted MAIT cells (data files and protocols can be found in Leng *et al*.^21^). THP‐1 cell RNA‐seq libraries were prepared using the NEBNext Ultra II Directional RNA Library Prep Kit for Illumina (New England BioLabs Ltd, Hitchin, UK) following the kit protocol. The libraries were then sequenced on Illumina NextSeq500 (Peter Medawar Building for Pathogen Research, Oxfordshire, UK) machine acquiring 75‐bp paired end reads from three replicates of each condition. The data were analyzed on Partek Flow Genomics Suite (Partek Ltd, Southampton, UK). Reads were aligned using the Star aligner and the hg19 reference genome. Raw counts were used to determine expression of prostaglandin receptor genes in MAITs and THP‐1 cells.

#### Flow cytometry

Cells were washed two times in PBS and then stained with near infrared (Invitrogen, Loughborough, UK) for 10 min in the dark at room temperature. Cells are then washed again in excess PBS and then fixed with 4% paraformaldehyde in PBS for 10 min followed by permeabilization in perm buffer (Invitrogen, Loughborough, UK) for a further 10 min. The cells were then stained with CD3‐e450/CD3‐PerCP‐Cy5.5; clone‐UTCH1/OKT3 (eBioscience, BioLegend, Cheshire/Berkshire, UK), CD8‐VioGreen; clone‐BW135/80 (Miltenyi Biotech, Working, UK), CD161‐PE; clone‐9‐1BB (Miltenyi Biotech, Working, UK), Vα7.2‐APC; clone‐3C10 (BioLegend, London, UK), IFNγ‐PerCP‐cy5.5; clone‐4 S.B3 (eBioscience, Cheshire, UK), TNF‐α‐PE‐Cy7; clone‐Mab11 (BioLegend, London, UK), CD69‐FITC/Pacific blue; clone‐FN50 (BioLegend, London, UK), granzyme‐B‐efluor‐450; clone‐MHGB05 (Invitrogen, Scotland, UK), CCL3‐FITC; clone‐REA257 (Miltenyi Biotech, Woking, UK), Furin‐AF647; clone‐222 722 (R&D, Oxfordshire, UK) and IL‐17F‐PE; clone‐SHLR17 (Invitrogen, Scotland, UK) in perm buffer for 20 min in the dark at room temperature. Unbound antibody was washed off with excess PBS and then the cells were resuspended in 100 μL. Data were acquired on the Miltenyi MACSQuant or MACSQuant X.

#### Surface staining of antigen presenting molecules

*Escherichia coli*–stimulated THP‐1 cells were cultured with titrations of ± PGD_2_ and PGE_2_ overnight. THP‐1 cells were washed two times with PBS and stained with Live/Dead near‐infrared stain for 10 min. The assay was split equally to stain one‐half with isotype antibodies. The cells were washed once again and then labeled with a cocktail of MR1‐PE; clone‐26.5 (BioLegend, London, UK), HLA‐ABC‐APC; clone‐W6/32 (eBioscience, Cheshire, UK) and HLA‐DR‐PE‐Cy7; clone‐L243 (BioLegend, London, UK) or MR1 isotype control, IgG2κα‐PE; clone‐MOPC‐173 (BioLegend, London, UK).

#### Analysis

Flow cytometry data were analyzed by FlowJo version 10.7.1 and 10.8.0. Dead cells were excluded prior to gating for single cells on forward scatter–area and side scatter–area. Next, we gated the CD3 and CD8 double‐positive events, followed by MAIT gating, CD161^++^ and Vα7.2^+^ double‐positive events. Total MAIT cells gated as CD3‐positive CD161^++^Vα7.2^+^ MAIT cells were analyzed. This population was then used to quantify percentage IFNγ‐, TNF‐α‐, CD69‐, GrB‐, CCL3‐, furin‐ and IL‐17F‐positive cells. Representative flow cytometry gating is shown in Supplementary figure 1.

#### Statistics

Graphs were drawn and statistical analyses were carried out using Graph Pad Prism, version 8. Bar graphs represent the standard error of the mean and a one‐way ANOVA, Šidák's multiple comparisons *t*‐test was used to determine statistical significance which is mentioned in the corresponding figure captions.

## CONFLICT OF INTEREST

The authors declare that the research was conducted in the absence of any commercial or financial relationships that could be construed as a potential conflict of interest.

## ETHICS STATEMENT

Blood samples were acquired from healthy volunteers from the NHS blood bank (Oxfordshire) through ethical approval by the Central Office for Research Ethics Committees (COREC, local research ethics committee Oxford; reference number: YH160247).

## FUNDING INFORMATION

This work has been funded by the Klenerman CLIP grant, P Klenerman WT investigator award, NIH U19, and PK NIHR Senior Investigator Award.

## AUTHOR CONTRIBUTIONS

**Hema Mehta:** Conceptualization; data curation; formal analysis; investigation; project administration; visualization; writing – original draft; writing – review and editing. **Irene Tasin:** Data curation. **Carl‐Philipp Hackstein:** Data curation; methodology. **Christian Willberg:** Conceptualization; supervision. **Paul Klenerman:** Conceptualization; funding acquisition; investigation; supervision; writing – review and editing.

## Data Availability

The data that support the findings of this study are available from the corresponding author upon reasonable request.
