# Supplementary material for: Prostaglandins differentially modulate mucosal‐associated invariant T‐cell activation and function according to stimulus
Source: Immunol Cell Biol. 2023 Feb 18;101(3):262–72. doi: 10.1111/imcb.12617 (PMC10152717; doi:10.1111/imcb.12617)
Supplement: Supplementary file 1 [file IMCB-101-262-s001.pdf]

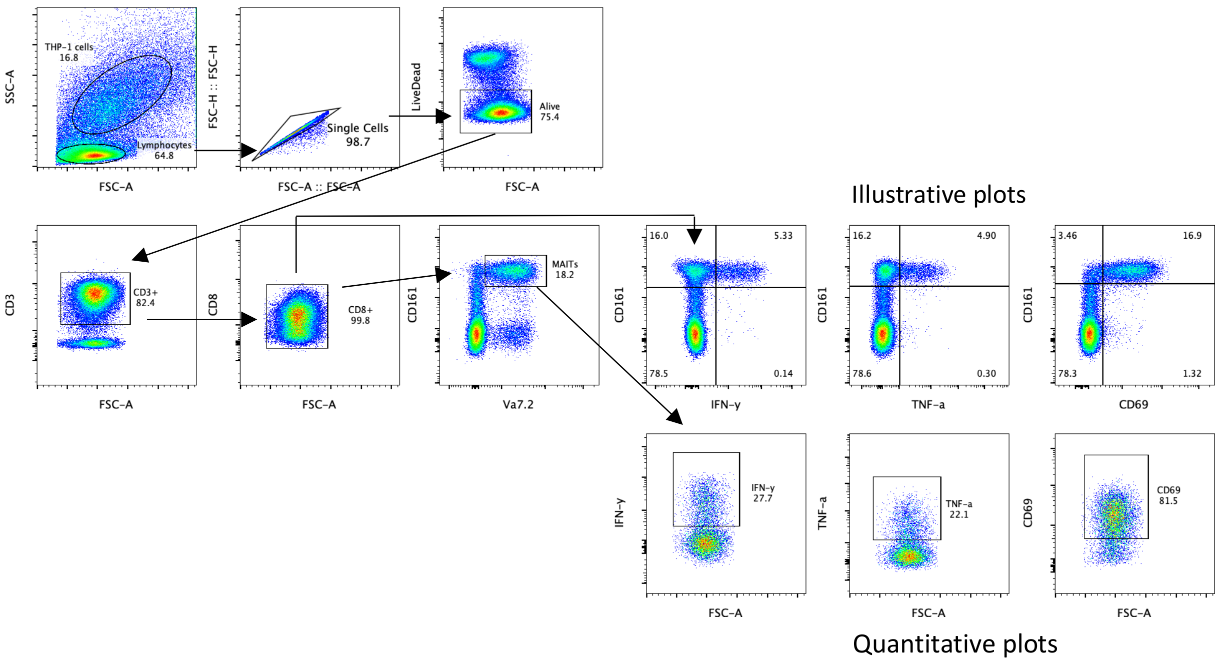

Supplementary figure 1 – Gating strategy – lymphocytes were gated using the FSC-A and SSC-A parameters, followed by single cell gating on the FSC-A vs FSC-H plot. Next, we gated on viable cells that were negative for the LiveDeadNIR viability dye. The next two gates selected the CD3 and CD8 T cells followed by MAIT cell gating using the CD161 vs Vα7.2 parameters. The MAIT cell population was then analysed for markers individually by looking at each of the parameters on the Y axis against the FSC-A or against CD161 as in the representative plots.

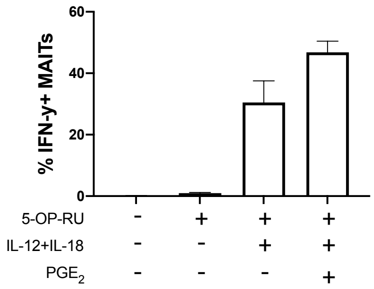

Supplementary figure 2 – IFN-y expression from PBMC-MAIT cells stimulated with 5-OP-RU and IL-12+IL-18, ± PGE_2 for 72 hours. IFN-y expression measured by intracellular staining and flow cytometry (n = 4).

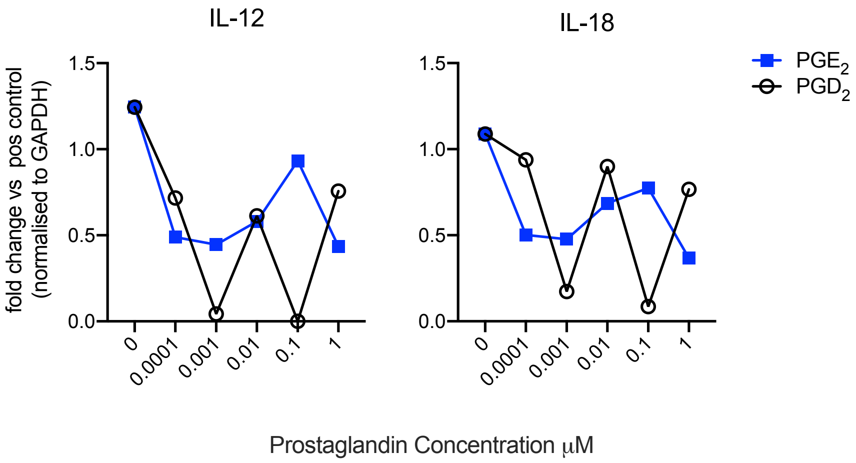

Supplementary figure 3 – IL-12 and IL-18 gene expression analysis in *E. coli* stimulated THP-1 cells, treated with prostaglandin titrations. Data presented as fold change relative to the untreated condition with prior normalisation to GAPDH.

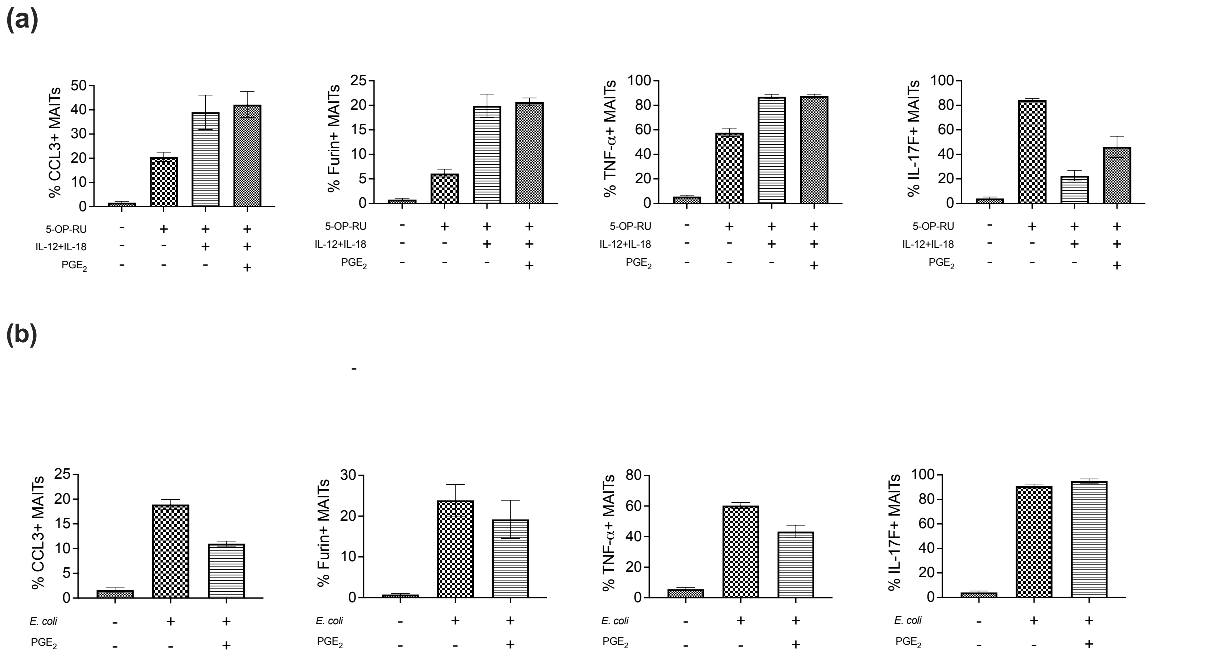

Supplementary figure 4 – Measurement of tissue repair factors from MAIT cells. Healthy donor PBMCs stimulated with 5-OP-RU and IL-12+ IL-18 **(a)** or *E. coli* **(b)** ± PGE_2 for 72 hours followed by flow cytometry analysis of CCL3, Furin, TNF-a and IL-17F.
